# Sexual and Psychosocial Functioning in Women with MRKHS after Neovaginoplasty According to Wharton-Sheares-George: A Case Control Study

**DOI:** 10.1371/journal.pone.0124604

**Published:** 2015-04-22

**Authors:** Katharina Leithner, Andrea Naderer, Dorothee Hartung, Clara Abrahamowicz, Johanna Alexopoulos, Katharina Walch, René Wenzl, Eva Hilger

**Affiliations:** 1 Department of Psychoanalysis and Psychotherapy, Medical University Vienna, Vienna, Austria; 2 Department of Obstetrics and Gynaecology, Divison of Gynaecology and Gynaecological Oncology, Medical University Vienna, Vienna, Austria; 3 Department of Neurology, Medical University Vienna, Vienna, Austria; Medical University of Vienna, AUSTRIA

## Abstract

**Background:**

Mayer-Rokitansky-Küster-Hauser syndrome (MRKHS) has a major impact on a woman’s psychological and sexual well-being. In most of the studies that addressed treatment techniques, postoperative sexual function was reported to be satisfactory; however, comparatively few studies have additionally provided an accurate analysis of psychosocial functions in MRKHS patients following different kinds of neovaginal treatment. This study is to evaluate sexual and psychosocial functioning after creation of a neovagina according to Wharton-Sheares-George in women with MRKHS.

**Methods:**

We performed a case-control-study using multiple measures to assess sexual and psychosocial functioning. Ten MRKHS patients and 20 controls of a University hospital and tertiary center for pediatric and adolescent gynecology were assessed. The follow-up assessment comprised 6 standardized questionnaires (Female Sexuality Function Index, FSFI; Patient Health Questionnaire, PHQ; Brief Symptom Inventory, BSI; World Health Organization Quality of Life Assessment, WHOQoL-BREF; Parental Bonding Instrument, PBI; and a German questionnaire on body image). The main outcome measures were sexual function, psychological status, quality of life, body image, and parental bonding styles.

**Findings:**

Sexual function, psychological status (including depressive and somatic symptoms), quality of life, and own-body experience were at least as good in operated MRKHS patients as in controls. In some measures (FSFI, PHQ-15, psychological domain of the WHOQoL-BREF, and BSI Positive Symptom Total), patients scored significantly better than controls. The results of the PBI indicated a close and sustainable mother-daughter-relationship in MRKHS patients.

**Conclusions:**

We found no evidence for an impairment of sexual or psychosocial functioning in patients after neovaginoplasty according to Wharton-Sheares-George. MRKHS may not necessarily compromise sexual and psychological well-being, provided that the syndrome is properly managed by a multidisciplinary team of health professionals.

## Introduction

The Mayer-Rokitansky-Küster-Hauser syndrome (MRKHS), also referred to as müllerian agenesis or müllerian aplasia, is a congenital aberration of the female genital tract, with an estimated incidence of 1 per 4.500–10.000 females. Due to an embryologic growth failure of the müllerian duct, the most common presentation is the absence or underdevelopment of the vagina, uterus, or both. Individuals with MRKHS have a normal female 46, XX karyotype, and the ovaries, which are separate in their embryologic origin, are regular in structure and endocrine function [1].

MRKHS affects a woman’s ability to menstruate, to engage in penile-vaginal intercourse, and to carry a pregnancy. Vaginal reconstruction (i. e. the creation of a neovaginal canal of adequate diameter and length to allow for sexual intercourse) is one major treatment goal in MRKHS patients and can be accomplished non-surgically with vaginal dilatation, or surgically [2,3]. Numerous techniques of surgical creation of a neovagina have been proposed, such as the Abbe-McIndoe operation (i. e. dissection of a space between the rectum and bladder, placement of a mold covered with a skin graft into the space, and diligent postoperative vaginal dilation) and its modifications, the Vecchietti procedure (using continuous dilation with an external traction device that is temporarily affixed to the abdominal wall, either performed by laparatomy or as a modification), the Davydov operation (involving dissection of the rectovesicular space with abdominal mobilization of a segment of the peritoneum, and attachment of the peritoneum to the introitus), sigmoidal coloplasty (i. e., vaginal replacement or creation of a vagina by grafting a segment of sigmoid), and further techniques using buccal mucosa, amnion, or other allografts [1,2,4].

Inherently, a woman’s psychological and sexual well-being may profoundly be impacted by this syndrome [5]. In most of the studies that addressed treatment techniques, postoperative sexual function was reported to be satisfactory; however, comparatively few studies have additionally provided an accurate analysis of psychosocial functions in MRKHS patients following different kinds of neovaginal treatment [6,7].

Our group has published the George modification of the Wharton-Sheares technique as an effective and safe surgical option for vaginal reconstruction [8]. This procedure is based on the technique according to Sheares [9], whereby a perineal skin flap is inserted as an isograft into the space tunnelled between the rectum and bladder (with the müllerian ducts used for orientation), and on the principle of Wharton, which states that epithelialisation of the new tunnel occurs both from the vaginal orifice and from the edges of the isografts if a vaginal mold is left undisturbed for 2–3 weeks. George modified the Sheares technique by forbearing from inserting the mucosal flap into the posterior surface of the newly created cavity. More recently, we reported the favorable anatomic and functional long-term results after this kind of neovaginal surgery [10].

In the present study, we performed an elaborated psychometric follow-up assessment in MRKHS patients who had undergone surgery according to Wharton-Sheares-George. Multiple measures were used to assess sexual function, psychological status, quality of life, body image, and parental bonding styles. Calculated scores of these measurements were compared to those of an age- and education-matched control group.

## Materials and Methods

MRKHS patients were recruited within the scope of their postoperative aftercare at the Division of General Gynaecology and Gynaecological Oncology (Department of Obstetrics and Gynaecology of the Medical University of Vienna). Out of 17 patients who had surgically been treated with neovaginoplasty according to Wharton-Sheares-George, 10 patients (mean age: 22.9 ± 5.70 yrs) were enrolled in the study. Seven women were not included because of the following reasons: interval of less than three months between surgical treatment and time of investigation (N = 3), refusal of participation due to distant place of residence (N = 1), and missing contact details (N = 3). In the MRKHS study group, mean age at time of diagnosis was 16.8 (±1.87; range: 15–21) years. Patients were investigated between September 2009 and February 2010. At the time of investigation, the operation had been 3 to 77 months ago. All patients had been offered psychological counseling before and after surgical treatment. Two patients had attended a single session with a psychotherapist prior to the operation. Five patients (50.0%) had been treated psychotherapeutically on a regular basis before surgery and 2 patients (20.0%) after surgery, respectively.

The control group consisted of 20 age- and education-matched female control subjects (mean age: 25.5 ± 4.21 yrs). Controls were recruited in the frame of their routine (preventive) evaluation at their gynaecologists in private practice from June 2010 to March 2012. None of these women had been diagnosed with any psychiatric disorder or gynaecological disease, nor did any of them exhibit psychiatric symptoms or had a severe somatic disorder at time of investigation. Socio-demographic data of patients and controls are presented in Table 1.

**Table 1:** Socio-demographic data of patients with MRKHS (N = 10) and controls (N = 20).

	Patients	Controls
**Marital status, N (%)**		
Married/cohabiting	1 (10)	3 (15)
Divorced/widow/single	6 (60)	9 (45)
Partnership	3 (30)	8 (40)
**Education level, N (%)**		
Compulsory school	1 (10)	2 (20)
Vocational school	2 (20)	4 (20)
High school	6 (60)	12 (60)
University	1 (10)	2 (10)
**Employment status, N (%)**		
Employed	5 (50)	7 (35)
Unemployed	1 (10)	12 (60)
Student	4 (40)	10 (5)

Both MRKHS patients and controls completed six well-validated self-report questionnaires to assess sexual function (Female Sexual Function Index, FSFI), psychological status (Patient Health Questionnaire, PHQ; and Brief Symptom Inventory, BSI), subjective quality of life (WHOQOL-BREF), body image (Fragebogen zur Beurteilung des eigenen Körpers, FBeK), and parenting behavior (Parental Bonding Instrument, PBI).

The FSFI [11] (German version [12]) assesses sexual function in women during the past 4 weeks. It consists of 19 items subdivided into six domains: desire, arousal, lubrication, orgasm, satisfaction, and pain. Full-scale scores range from 2.0 to 36.0, with high scores indicating better sexual function. Usually, total FSFI scores less than 23 are considered “poor”, “good” when ranging between 24 and 29, and “very good” when ≥ 30. Three patients without sexual activity within the past 4 weeks prior to the investigation were not included in the FSFI evaluation.

The PHQ [13] (German version [14]) is a shortened version of the Prime-MD [15], which allows the detection of frequent psychiatric disorders in primary care. For the purpose of this study, three continuous subscales of the PHQ (PHQ-9, PHQ-15 and the PHQ-stress score), were used. The PHQ-9 (total score: 27) measures depression severity, and the PHQ-15 (maximum score: 30) somatization symptoms, respectively. The PHQ total stress score (maximum score: 20) assesses psychosocial stress factors. High scores indicate high impairment within these domains.

The BSI is a questionnaire measuring psychological distress [16] (German version [17].). It contains 53 questions in which participants rate the extent to which they have been bothered (0 = "not at all" to 4 = "extremely") in the past week by various symptoms. Amongst others, the BSI includes 3 global indices that capture global psychological distress. The “Global Severity Index” (GSI) measures basic psychological impairment, the “Positive Symptom Distress Index” (PSDI) measures the intensity of the answers, and the “Positive Symptom Total” (PST) represents the number of symptoms with impairment. High scores of these indices indicate a high degree of psychological impairment.

The WHOQOL-BREF is a short version of the World Health Organization Quality of Life Assessment [18] (German version [19]). It consists of 26 items belonging to four domains: “Physical Well-Being”, “Psychological Well-Being”, “Social Relationships and Environment”, and “Global value”. Scores ranging from 0 to 5 points can be assigned to each item (final domain scores: 0 to 100). The higher the scores are, the better the perceived quality of life is.

The FBeK [20] consists of 52 items capturing the subjective body image. Each item is a statement which can be agreed (1 point) or negated (0 points). Items are clustered into 4 scales: “Attractiveness/ Self-Confidence” (scale 1; 15 items), “Accentuation of Physical Appearance” (scale 2; 12 items), “Insecurity/ Concern” (scale 3; 6 items), and “Physical/ Sexual discomfort” (scale 4; 13 items). Thus, maximum scores to be achieved in each scale are 15, 12, 13, and 6 points, respectively. High scores indicate high satisfaction with the own body (scale 1), an intensive preoccupation with the importance of physical appearance (scale 2), high insecurity (scale 3), and strong physical and sexual discomfort (scale 4). The internal consistency was tested and found to be sufficient, with Cronbach’s α between 0.69 and 0.85. Convergent validity was shown to be satisfactory. Table 2 shows examples of items of each scale.

**Table 2:** “Fragebogen zur Beurteilung des eigenen Körpers” (FBeK): A tool to evaluate subjective body image.

Scales	Number of Items	Item Example	Rating (points)
**Scale 1: Attractiveness/ Self-Confidence**	15	“I am satisfied with my appearance.”	yes (1), no (0)
**Scale 2: Accentuation of Physical Appearance**	12	“I often look into the mirror.”	yes (1), no (0)
**Scale 3: Insecurity/ Concern**	13	“I often feel clumsy.”	yes (1), no (0)
**Scale 4: Physical/ Sexual discomfort**	6	“I am satisfied with my sexual experience.”	yes (1), no (0)

The PBI [21] (German version [22]) measures parental styles. The measure is retrospective, thus adults complete the measure (for both mothers and fathers separately) for how they remember their parents during their first 16 years. The 25 items, assessing the domains of “care” (12 items) and “overprotection” (13 items), are scored on a 4-point scale (“Very like” to “Very unlike”). An example of an item scored on the “care” domain is “could make me feel better when I was upset;” an item scored on the “overprotection” domain is “did not want me to grow up.” Scores below 27/ 24 indicate low maternal/ paternal care, and scores above 13.5/ 12.5 indicate high maternal/ paternal overprotection.

The study protocol has been approved by the local Ethics Committee of the Medical University of Vienna. All participants gave written consent prior to any investigation.

### Statistical Analysis

To evaluate differences between the control and patients groups, a comparison between paired samples was carried out. Two subjects of the control group were each matched with one subject of the case group. To obtain an averaged control subject for each MRKHS patient, we calculated the means of each pair of age- and education matched controls. Consecutively, a paired-sample t-test was applied, and a Wilcoxon signed-rank test when appropriate. Participants without sexual activity in the past months were excluded from the FSFI evaluation. For all analyses, the significance threshold was set to p ≤ 0.05 using two-tailed significance levels. All statistical analyses were carried out using IBM SPSS Statistics 19 software (IBM Corp., Armonk, NY, USA).

## Results

As previously reported, MRKHS patients exhibited satisfactory sexual function as indicated by high mean FSFI scores (29.90 ± 4.30) [10]. When compared to the control group, controls (n = 20) had marginally, however significantly, lower scores on the FSFI (mean: 24.10 ± 6.40; p =. 049).

On the PHQ, patients with MRKHS showed significantly less somatic symptoms than controls (PHQ-15: patients: 3.50 ± 2.72; controls: 5.65 ± 3.42; p =. 014). Depressive symptomatology (PHQ-9) and psychosocial burden (PHQ-stress score) were not significantly different between groups (p =. 055, and p =. 675, respectively). One woman out of the control group (but none of the patient group) reached a PHQ-score ≥10, suggesting the presence of a major depressive disorder.

When comparing BSI scores between MRKHS patients and controls, patients had significantly lower “Positive Symptom Total” scores indicating less psychological impairment than controls (patients: 10.90 ± 9.29; controls: 18.50 ± 11.39; p =. 017). Severity of symptoms (as measured by the “Positive Symptom Distress Index”) and basic psychological impairment (as measured by the “Global Severity Index”) were not different between groups (p =. 161, and p =. 076, respectively).

Regarding the results of the WHOQoL-BREF, no group differences were found on the domains “Physical health” (p = 0.89), “Social relationships” (p =. 305), “Environment” (p =. 080), and “Global” (p =. 105). Within the domain “Psychological”, MRKHS patients scored significantly higher than controls (patients: 80.33 ± 10.27; controls: 68.75 ± 14.01; p =. 004).

With respect to own-body-experience, as assessed by the FBeK, in none of the four scales (“Attractiveness/ Self-Confidence”; “Accentuation of Physical Appearance”; “Insecurity / Concern”, “Physical/ Sexual discomfort”) a significant difference was observed between patients and controls. Calculated scores of the FSFI, PHQ, BSI, and WHOQoL-BREF are provided in Table 3.

**Table 3:** Sexual and psychosocial functioning in patients with MRKHS (N = 10) and controls (N = 20).

	Patients	Controls	p-value
**Sexual function**			
FSFI total score^a^	29.90 (4.30)	24.10 (6.40)	.*049*
**Psychological status (BSI, PHQ)**			
BSI Global Severity Index	0.35 (0.33)	0.53 (0.43)	.161
BSI Positive Symptom Distress Index	1.70 (0.56)	1.35 (0.35)	.076
BSI Positive Symptom Total	10.90 (9.29)	18.50 (11.39)	.*017*
PHQ-9 (depression severity scale)	3.00 (2.79)	5.55 (4.12)	.055
PHQ-15 (somatic symptoms severity scale)	3.50 (2.72)	5.65 (3.42)	.*014*
PHQ- total (psychosocial) stress score	3.80 (3.52)	4.30 (4.23)	.675
**Quality of Life (WHOQOL-BREF)**			
Physical health	88.93 (9.89)	79.11 (14.02)	.089
Social relationships	78.32 (15.31)	72.92 (19.66)	.305
Psychological	80.33 (10.27)	68.75 (14.01)	.*004*
Environment	83.86 (10.72)	75.32 (11.56)	.080
Global	90.00 (16.46)	78.75 (15.23)	.105
**Own-body-experience (FBeK)**			
Attractiveness/ Self-confidence	13.1 (2.60)	11.85 (3.53)	.099
Accentuation of Physical Appearance	8 (2.45)	8.55 (1.76)	.448
Insecurity / Concern	3.4 (2.76)	3.85 (2.62)	.552
Physical/ Sexual discomfort	1.4 (1.07)	1.6 (1.5)	.689

Note: Values are given as means (SD); p-values in italic indicate statistical significance

Abbreviations: FSFI: Female Sexual Function Index; PHQ: Patient Health Questionnaire; BSI: Brief Symptom Inventory; WHOQOL-BREF: World Health Organization Quality of Life Assessment; FBeK: Fragebogen zur Beurteilung des eigenen Körpers; PBI: Parental Bonding Instrument

^a^ 3 patients without with sexual activity 4 weeks prior to the investigation were excluded from the FSFI calculation

Analysis of the results of the PBI revealed that MRKHS patients perceived their mothers as significantly more caring than the control group did (PBI subscale “care”/ maternal style: patients: 32.3 ± 4.21; controls: 26.4 ± 7.45; p =. 036). With respect to the paternal “care” subscale, and both the maternal and paternal “overprotection” subscales, no significant group differences were found (see Table 4).

**Table 4:** Parental bonding in patients with MRKHS (N = 10) and controls (N = 20).

	Patients	Controls	p-value	Patients	Controls	p-value
PBI subscales	Maternal			Paternal		
Care	32.3 (4.21)	26.4 (7.45)	.*036*	26.4 (7.71)	25,3 (7.84)	.414
Overprotection	12.6 (10.8)	10.9 (8.27)	.616	8.8 (6.81)	11.0 (7.82)	.430

Note: Values are given as means (SD); p-values in italic indicate statistical significance

PBI: Parental Bonding Instrument

## Discussion

In this study, we provide an analysis of both sexual and psychosocial function using 6 standardized questionnaires in 10 MRKHS patients after neovaginal surgery according to Wharton-Sheares-George. Twenty females without MRKHS served as a control group.

As indicated by a mean total FSFI score of 29.90 ± 4.30, sexual function was rated good in operated patients, with a marginal, yet significant difference in favour of the patient group compared to controls. With respect to psychological status (including depressive and somatic symptoms as assessed by the PHQ, and global psychological distress as measured by the BSI), we found no evidence for a significant impairment in our MRKHS sample. Of note, MRKHS patients showed even less somatic symptoms (PHQ-15) than controls, and had lower scores on the BSI global index “Positive Symptom Total”, indicating less psychological impairment than controls. Overall quality of life was comparable between groups, with no statistical significance in 4 out of 5 domains of the WHOQoL-BREF. Within the domain “Psychological”, MRKHS patients scored even higher than controls. Body image (including sexual comfort and feelings of shame), as assessed by the FBeK, was not different between patients and controls. Finally, results of the PBI indicated that patients perceived their mothers as significantly more caring as controls did.

Our study suffers some limitations, such as the use of self-questionnaires with its inherent shortage in terms of external objectivity, the varying intervals between the surgical intervention and the time of investigation, and the lack of a baseline assessment (i. e. completion of the same questionnaires before surgery). Thus, no definite conclusions can be drawn whether an improvement of sexual and psychosocial function (or a lack thereof) is directly related to the surgical procedure. Moreover, the sample size (though fair in light of the rare nature of the syndrome and this kind of surgery) may have compromised statistical power. The fact that only 10 out of 17 patients who had undergone vaginoplasty according to Wharton-Sheares-George could be enrolled in the study, may limit the generalizability of our findings. Potential strengths of our study are the use of numerous rating instruments with proven reliability and validity of both their original and German versions, the inclusion of a well-matched control group and the fact that this is the first study assessing both sexual function and psychological status after vaginoplasty according to Wharton-Sheares-George.

Previous studies that operationally defined and explicitly evaluated sexual function after neovaginoplasty using different surgical techniques have yielded comparable results. With the exception of one report, in which sexual function after the laparoscopic Davydov procedure (N = 6) was significantly impaired compared with controls [23], postoperative sexual function was consistently satisfactory throughout studies. Giannesi et al. [24] obtained an average FSFI of 26.5 ± 5.6 in 28 patients treated with the laparoscopic Davydov procedure. With respect to the Vecchietti procedure and its laparoscopic modifications, mean FSFI scores were reported to be 30.1 ± 3.8 (N = 23) [25], and 29.0 ± 3.2 (N = 27) [26], respectively. In a series of 5 patients treated with amnion vaginoplasty, mean FSFI was 30.0 ± 6.9 [27]. Other authors evaluated vaginoplasty using tissue-engineered biomaterial graft (N = 24) [28] or laparoscopic peritoneal vaginoplasty (N = 31) [29] and reported an average FSFI of 26.7 ± 3.5, and 26.09 ± 4.82, respectively. In none of these studies, a significant difference was found between patients and controls. Similar figures (i. e. FSFI scores ranging between 28.0 and 30.0) can be inferred from a number of other studies evaluating the sexual results of surgical techniques such as rectosigmoid vaginoplasty or amnion vaginoplasty [30–32], but no controls were included in these studies.

Findings of prior studies investigating psychological well-being are inconsistent, and comparison of the results is hampered due to the use of different psychosocial assessment methods and the heterogeneity of investigated MRKHS samples with respect to the treatment performed. Some authors reported significant impairments in various domains of psychosocial functioning in MRKHS patients, such as disturbances of body image and self-esteem [33–35], elevated depression rates [7,31,36] and a profound lack of sexual confidence [37,38]. Others found an alleviation of psychological distress related to the creation of a neovagina [39–41]. There are, however, only 3 studies that used multiple well-validated rating instruments for the assessment of psychosocial functions. Heller-Boersma et al. [36] compared 66 MRKHS patients with 31 control subjects and found MRKHS patients to have significantly more pathological scores on scales measuring phobic anxiety and psychoticism (and a similar trend for depression and anxiety), but no information about treatment was provided in this study. Likewise, in as study recently published by Fliegner et al. [7], depression rates were slightly elevated and apprehensiveness in sexual situations was a major source of distress in 49 MRKHS patients with and without neovaginal treatment. Another study comprehensively investigating psychosocial functioning in 40 patients after colovaginoplasty found no overrepresentation of psychosocial problems or depressive symptoms in patients compared to controls [6]. Thus, available data do currently not allow the conclusion that a distinct surgical procedure may have a particular positive or negative impact on psychosocial outcome. However, our results are well in line with those of Gatti et al. [6], suggesting that neovaginal surgery does not inherently compromise psychosocial functioning in women with MRKHS.

Of note, there are almost no studies reporting the use of standardized quality of life questionnaires in MRKHS patients; instead, “quality of life” has most often been indirectly been derived from primary outcome measures such as sexual function or self-esteem. There is, however, one study in which the WHOQoL-BREF was administered in 28 MRKHS patients after surgical or non-surgical neovaginal treatment [42]. As in our sample, no evidence of an impairment of quality of life was found in patients with MRKHS.

Finally, results of the PBI indicated that MRKHS patients perceived their mothers as significantly more caring than controls did, suggesting a particular close and/ or sustainable mother-daughter-relationship in our patients. It has to remain unclear to what extent this finding may have contributed to the good overall psychological outcome, since the sample size of this study did not allow reliable calculations of a correlation between PBI scores and other psychometric measurements. Moreover, we cannot rule out that this finding has emerged by chance due to the small sample size. However, the mother-daughter relationship is known to have crucial impact on the development of female sexuality, body image, and feelings of bodily integrity [43]. Of interest, there is one qualitative study based on 8 MRKHS patients suggesting that a stable mother-daughter-relationship, based on trust and esteem, may positively influence postoperative results in patients with MRKHS [44].

## Conclusions

We found sexual function and psychological status to be without any pathological findings in patients after neovaginoplasty according to Wharton-Sheares-George. MRKHS patients scored even better than controls in some of the measures such as the FSFI, PHQ-15, the psychological domain of the WHOQOL-BREF, and the BSI “Positive Symptom Total”. In light of the small sample size, this finding should not be over interpreted, however, one may speculate that in some patients, the creation of a neovagina with consecutive sexual rehabilitation results in a kind of psychological “rebound phenomenon” with overcompensated feelings of well-being and self-esteem.

Our results support the assumption that MRKHS may not compellingly interfere with psychological and sexual well-being, provided that the syndrome is adequately managed by experienced health professionals. Future studies including pre- and post surgery evaluations may further elucidate the differential sexual and psychological outcome of the several methods of neovaginal surgery.

In any case, the management of patients with MRKHS should focus on both, the need for psychological support, and the necessity to anatomically manage the anomaly. Irrespective of the surgical strategy chosen, the management of MRKHS patients should probably be interdisciplinary. Future research should also assess the need and acceptance of psychological support for women with MRKHS before and after surgery.
